# The sense of hearing in the Pacific oyster, *Magallana gigas*

**DOI:** 10.1371/journal.pone.0185353

**Published:** 2017-10-25

**Authors:** Mohcine Charifi, Mohamedou Sow, Pierre Ciret, Soumaya Benomar, Jean-Charles Massabuau

**Affiliations:** 1 University of Bordeaux, EPOC, UMR 5805, Arcachon, France; 2 CNRS, EPOC, UMR 5805, Talence, France; 3 Unit of Research on Biological Rhythms, Neuroscience and Environment, Faculty of Science, Mohammed V-Agdal University, Rabat, Morocco; Bigelow Laboratory for Ocean Sciences, UNITED STATES

## Abstract

There is an increasing concern that anthropogenic noise could have a significant impact on the marine environment, but there is still insufficient data for most invertebrates. What do they perceive? We investigated this question in oysters *Magallana gigas* (*Crassostrea gigas*) using pure tone exposures, accelerometer fixed on the oyster shell and hydrophone in the water column. Groups of 16 oysters were exposed to quantifiable waterborne sinusoidal sounds in the range of 10 Hz to 20 kHz at various acoustic energies. The experiment was conducted in running seawater using an experimental flume equipped with suspended loudspeakers. The sensitivity of the oysters was measured by recording their valve movements by high-frequency noninvasive valvometry. The tests were 3 min tone exposures including a 70 sec fade-in period. Three endpoints were analysed: the ratio of responding individuals in the group, the resulting changes of valve opening amplitude and the response latency. At high enough acoustic energy, oysters transiently closed their valves in response to frequencies in the range of 10 to <1000 Hz, with maximum sensitivity from 10 to 200 Hz. The minimum acoustic energy required to elicit a response was 0.02 m∙s^-2^ at 122 dBrms re 1 μPa for frequencies ranging from 10 to 80 Hz. As a partial valve closure cannot be differentiated from a nociceptive response, it is very likely that oysters detect sounds at lower acoustic energy. The mechanism involved in sound detection and the ecological consequences are discussed.

## Introduction

There is an increasing concern that anthropogenic noise could have a significant impact on the marine environment. It has been shown that man-made noise has a deleterious effect on marine mammals, fish and cephalopods (see Peng et al. [1] for a review), but there is still insufficient data on the effect of noise on most invertebrates [2]. More generally, there is insufficient data on the ability of invertebrates to detect either water-borne or substrate-borne vibrations. What are their sound perception capacities? We addressed this question in a filter-feeder, the Pacific oyster *Magallana gigas (Crassostrea gigas*), a bivalve mollusc. It is plentiful along various coasts and is the object of major aquaculture efforts in numerous countries. In aquatic animals, the ability to detect acoustic signals—either transmitted through the water column or via the substrate where they live—is of paramount importance, as acoustic signals have unique advantages by comparison to visual and chemical stimuli: they travel rapidly, and one can detect them independent of the light levels and current direction [3].

Sound detection in invertebrates has been largely studied on crustaceans, which use sound vibrations for communication. By means of behavioural and electrophysiological techniques, thresholds have been determined, providing support for low-frequency sensitivity [4–8]. In Mollusca, studies on cephalopods have reported behavioural and physiological responses to waterborne sound stimuli at low frequencies [9–12]. In both groups the particle motion component of a sound wave, rather than the pressure, is likely to be what they perceive [7, 11,12]. In contrast, little is known about sound detection and sensitivity in bivalve molluscs despite their importance to the marine ecosystem.

To summarize, Mosher in 1972 [13] induced burrowing behaviour in the Baltic clam or Baltic tellin, *Macoma balthica*, by stimulating the wall of its experimental tank. In 1995, Ellers [14] performed an elegant study on a related bivalve, the digger boy or swatch-riding clam, which lives on sandy beaches of the East Coast of the USA. He demonstrated its ability to detect vibrations produced by the waves and to move up and down along a beach with the rising and falling tides. In 2005, Zhadan [15] reported that a special organ in pectens, the abdominal sense organ, is sensitive to water vibrations. Finally, in 2015 Roberts et al. [16] studied the sensitivity of the blue mussel, *Mytilus edulis*, to substrate-borne vibrations and demonstrated their sensitivity in the range of 5–400 Hz. Vazzana et al. in 2016 [17], working on the mussel *M*. *galloprovincialis*, reported an absence of behavioural reactions to sweep tones 0.1–60 kHz but significant changes of various biochemical parameters in their haemolymph, from 0.1–5 kHz. Peng et al. in 2016 [18], studying digging behaviour and gene expression in razor clams, showed that a white noise (at ≈ 80 and 100 dB re 1 μPa) induced deeper digging and changes in the expression of metabolism-related genes.

To determine whether an animal possesses the sense of hearing is a matter of definitions. If hearing is a response to the pressure component of sound in the ambient environment using specialized organs as ears, bivalve molluscs as many other animals do not hear. If according to Popper et al. [6] there is a need for a gas-filled space to sense pressure changes, again they cannot hear. However, Pumphrey [19] gave an alternative definition. For him, “Hearing is the reception of vibratory stimuli of any kind and nature, provided that the sound source is not in contact with the animal’s body”. In addition, Ladich and Fay [20] report that “hearing is the act of perceiving sound, a sensory function that involves the entire organism’s behaviour… which can only be measured using behavioural methods”.

In the present report on oysters, we chose group reactions of rapid valve closure as endpoints of behavioural responses to various sound frequencies at different acoustic energies. Indeed, a wide and resting opening status in unrestrained and settled bivalve molluscs is an index of their welfare [21–23]. In contrast, a rapid decrease of valve opening is a major way to protect their soft body when they detect a threat or whenever they are under special stress or pressure [22]. The objective of the study was to describe the hearing capacities of the Pacific oyster *M*. *gigas*, including its hearing range and sensitivity. The ecological consequences are discussed in terms of sound sources, including natural sources and noise pollution that contribute to their auditory environment.

## Materials and methods

All experiments were performed using 18-month-old Pacific oysters, *M*. *gigas* (diploid), at the Marine Station of Arcachon. They were purchased from local oyster farmers in the Bay of Arcachon, France and were chosen a priori according to their shell length (≈ 70–75 mm). This area has sporadic traffic noise mostly composed of recreational and fishing boats (6-19m). Two different groups of 16 oysters were studied in April-May 2015 (group A, n = 16) and September 2015 (group B, n = 16). All research detailed in this study complied with French law.

### Experimental design

**Fig 1:**
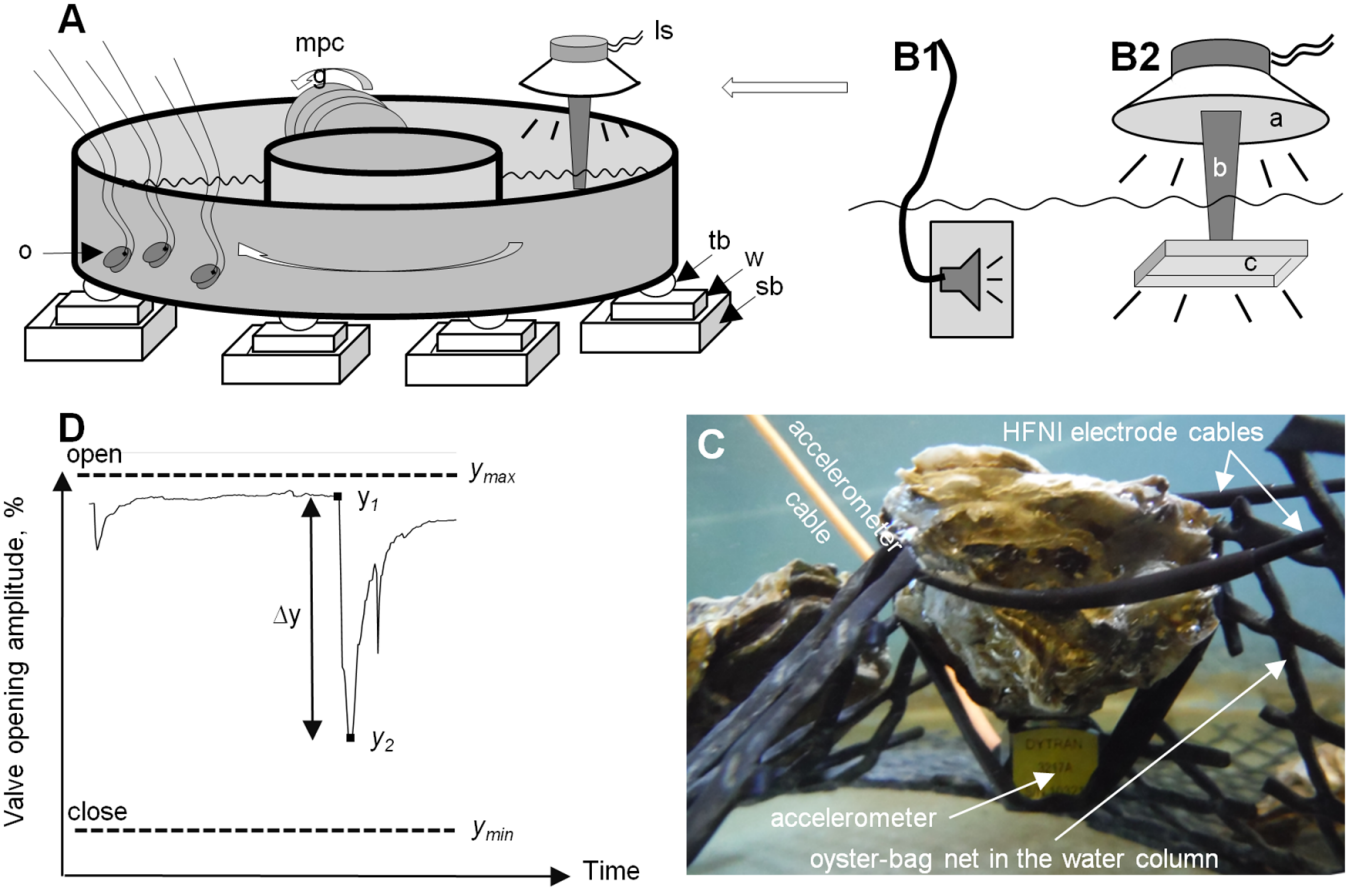
Experimental set-up and quantification of oyster response. A, schematic view. Is, loudspeaker position; mpcg, multiplate current generator; o, oysters equipped with electrodes; tb, tennis ball; w, wooden board; sb, sandbox; tb, w and sb compose a vibration absorber. B1, commercial loudspeaker to produce tones at frequencies from 80–20000 Hz; B2, laboratory-made loudspeaker for frequencies from 10–80 Hz. D, typical valve closure response and measured values: y_max_, daily maximum VOA (valve opening amplitude); y_min_, daily minimum VOA; y_1_, VOA prior to valve closing; y_2_, minimal VOA during response; Δy, amplitude of the response expressed in %, see the Materials and methods section. C, set-up for shell acceleration measurement.

The experiments were performed in a 350-L raceway (external diameter 2 m; internal diameter 1 m; depth 0.15 m; see Fig 1A). The set-up was an open flow system with a renewal rate of 250 L∙h^-1^. Oysters were continuously provided with unfiltered seawater directly pumped from the Bay of Arcachon, and dehydrated phytoplankton was added daily at 9 a.m. local time. Normoxic water flow was piped in underwater to prevent collision with the surface and decrease background noise. The water was maintained at a temperature of 15.0 ± 0.5°C, a salinity of 28-32/1000 and a pH of 7.8–8.0. Water current velocity deeply influences the ventilatory activity of filter feeders (see [24], for example) and it was accordingly decided that the water current would be kept constant throughout the experiment. To reduce noise production, the current was generated by a laboratory-made multiple-plate water current generator entrained by a motoreductor (MDP, France). The speed was constant, maintained at the desired value using a FIRST-DC-1C control card (MDP, France) controlled by LabVIEW (National Instrument). The near-bottom current was ≈ 5–8 cm s ^−1^, which was representative of speeds occurring in situ and reported at Eyrac Pier in front of the Marine Station of Arcachon, France [25]. The water level was constant. The photoperiod was artificially maintained constant above the oysters (L:D 12:12; irradiance above the oysters was ≈ 30 μE m^−2^ s^−1^ during the light period and ≈ 1 μE m^−2^ s^−1^ during the dark period, Biospherical Instruments Inc., San Diego, California, USA; neon light MASTER TL-D Xtra 36W/865 1SL, Philips, France). The flume was isolated from external vibrations using vibration-absorbing supports (from top to bottom: tennis balls, wooden boards and sandboxes, Fig 1A) in a room with no external light source. The rest of the room was not illuminated, and the equipment required for sound and behavioural acquisition was placed at a distance to limit external disturbance during and between trials. Oysters equipped with electrodes, see below, were acclimatized to the experimental set up for at least 10 days before any experiment. They were not lying on the bottom of the flume but on an empty oyster bag (mesh size 1.5 cm) in the water column.

### Sound generation and recordings (frequency, sound pressure and acceleration)

Oysters were exposed to different pure tones in order to assess the frequencies and acoustic energy levels that induce behavioural responses. The sound stimuli were created in Cool Edit Pro (version 2.0, Syntrillium Software Corporation, USA) using the tone generation tool (WAV format, sampling rate: 44.1 KHz, 16-bit resolution). Each track of 180 sec pure tone included a 70 sec fade-in (a gradual increase in the level of the audio signal) and no fade-out (no gradual decrease in the level of the signal). The tracks were played with a computer connected to an amplifier (model: AA-5810, AKAI Electric Co. Ltd., Japan) through two different underwater speakers depending on the sound stimulus: a commercial underwater loudspeaker and a laboratory-made loudspeaker. The commercial loudspeaker (model: US-0130, Randson Public Address, France) produced signals in the frequency range 80 Hz to 20 kHz (Fig 1B1). It was attached to a plate weighing 3 kg to increase its inertia and suspended in the water column using rubber bands, without direct contact with the bottom or the sides of the flume.

**Fig 2:**
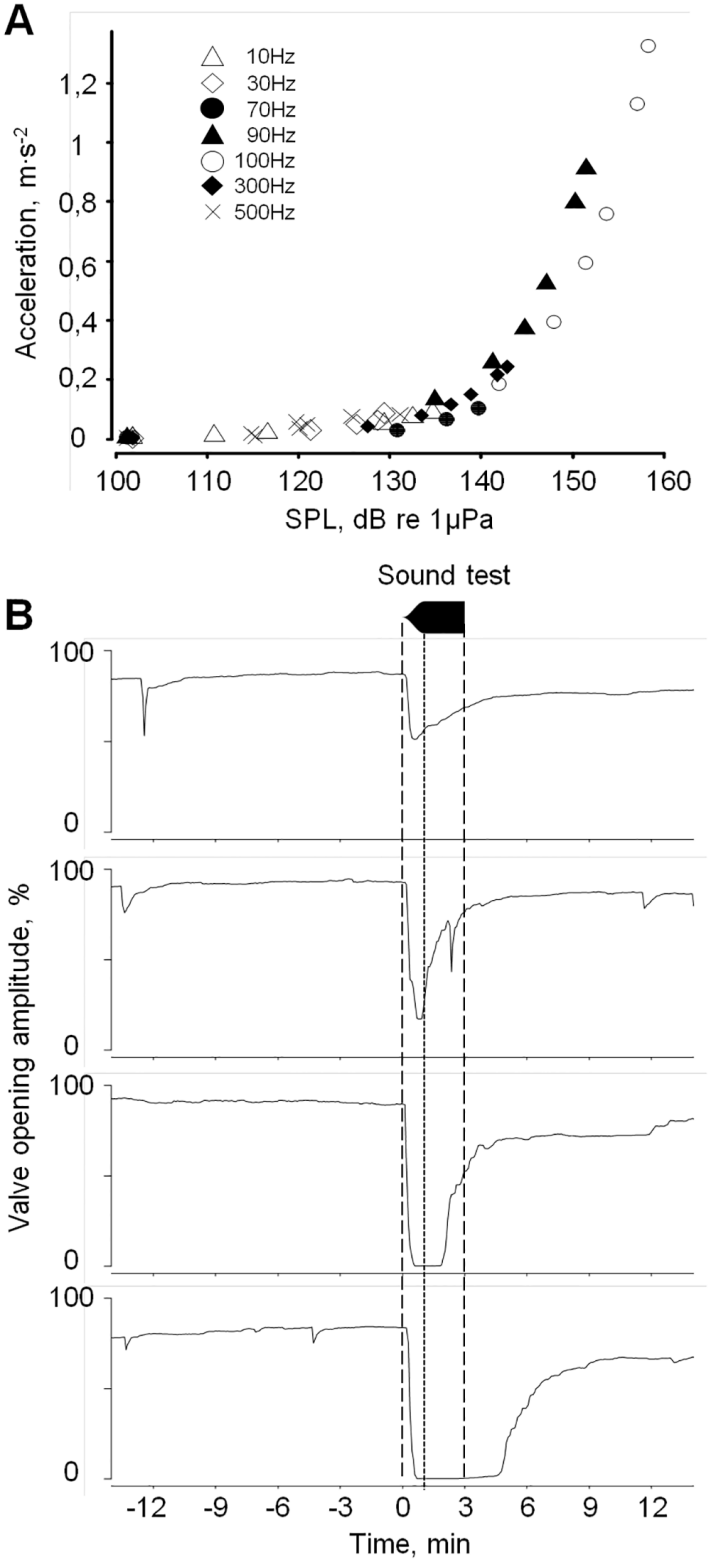
Typical oyster responses to 3 min of pure tone (100 Hz). A, relationship between shell acceleration and sound pressure level at various frequencies. B, from top to bottom, waveform and typical responses ranging from minimal to maximal responses as a function of time. Dashed lines, onset and offset of the stimulus; dotted line, end of the fade-in period; n = 4 individuals.

The laboratory-made underwater loudspeaker (Fig 1B2) was used to produce signals from 10 to 80 Hz. It was made from a 38 cm diameter loudspeaker with a mid/bass driver, impedance 4 ohms, 200 W rms (where rms is root mean square). An empty plastic cylinder (length 36 cm, diameter 7 cm) was glued on the loudspeaker’s membrane, and a rigid plastic plate (length, 25 cm; width, 18 cm; edge, 3 cm; thickness, 4 mm; 150 g) was glued at its end. In use, the body of the loudspeaker was lying on thick foam gaskets on the edge of the flume. Only the rectangular plastic plate was underwater. The sound was recorded at the level of the oysters using a broad-band hydrophone with an internal buffer amplifier (model: H2a-XLR, sensitivity -180 dB re 1 V/μPa, useful range: 10 Hz to 100 kHz, Aquarian Audio Product) linked to a voltage amplifier (Mx34c, Rolls Corporation, USA) and recording software (WaveLab v4.0, Steinberg Media Technologies). In a preliminary experiment, an accelerometer (Dytran underwater accelerometer, model 3217A-10, frequency range 1–10000 Hz, sensitivity 97 mV/g from 10–1000 Hz, weight 5 grams, size 12.7 x 12.7 mm) with 2 amplifiers in series (1 M28 IEPE, gain x1; 1 laboratory-made amplifier, gain x 100) was used to determine oyster shell accelerations at various sound pressure levels (SPLs) and frequencies. The analysis was performed on 5 oysters (shell length 70–75 mm, weight 72.4 ± 10.7 g; m ± sd) at different places on the oyster bag. On each animal, a flat surface was prepared on the lower cupped valve and the accelerometer was firmly secured to this surface with electrical tape (Fig 1C). There were no significant differences among the 5 oysters nor any based on position on the bag. For each frequency, the relationship between sound pressure level in the water column and shell acceleration was calculated and is shown in Fig 2A. The general relationship was y = e^(ax + b)^. At 10 Hz, a = -14.22, b = 0.087; 30 Hz, a = -19.9, b = 0.134; 70Hz, a = -21.74, b = 0.14; 90 Hz, a = -19.09, b = 0.13; 100 Hz, a = -18.48, b = 0.12; 300 Hz, a = -18.28, b = 0.12; 500 Hz, a = -11.24, b = 0.067. For technical reasons, it was not possible to measure acceleration for each oyster during the sound exposure runs, these curves were used to derive acceleration from SPL recordings. Shell acceleration and sound pressure level are expressed in rms for all measurements. The recording files were digitized at a 44.1 kHz sampling rate (16-bit resolution) and calibrated using pure sine waves played with a.wav player and measured at the output of the voltage amplifier with an oscilloscope (model: DSO-X 3012A, Agilent Technologies). No test was taken into account from 700–1000 Hz and 8–10 kHz due to loudspeaker technical restrictions (insufficient acoustic energy).

The background noise in the experimental flume was recorded with and without current. With the current generator running, the background sound pressure level was 101 ± 8 dBrms re 1 μPa in the water column and the reference acceleration was 0.003 ± 0.001 m∙s^-2^ on oyster shells (n = 15 records). The background noise was also recorded in the Bay of Arcachon in February (Eyrac Pier, latitude 44.66°, longitude -1.16°) using the same hydrophone. Each recording was 20 sec in duration, taken at a depth of 2 m (sea state 2) and without any boats (oyster farmers or coastal trawlers) in sight. The spectral levels of the recordings were analysed using a written script in R [26].

### Sound treatment protocol

Although pure tones are unlikely to occur often in the field, an understanding of oyster responses to them is fundamental to characterize their sound perception ability. The 1^st^ series of sound tests was performed on group A. During a single day, a sound test (1 pure tone, duration 3 min) was performed every 30 min according to the following sequence of frequencies: 90, 100, 200, 300, 400, 500, 600, 700 and 800 Hz. This protocol was repeated on 10 consecutive days. The 2^nd^ series of sound tests was performed on group B following the same experimental procedure but for frequencies ranging from 10–80 Hz. Over the experimental period, the oysters presented a diurnal behaviour pattern. All trials were conducted under light conditions.

### Behavioural analysis by valve activity recording

Valve activity was studied using high-frequency noninvasive valvometry. The HFNI valvometer is a high-frequency, noninvasive (HFNI) biosensor employed to monitor the valve behaviour of bivalve molluscs. It is a new-generation technique enabling the online study of the behaviour of bivalve molluscs in a lab or in their natural habitat, without interfering with normal behaviour.

Lightweight electromagnets, designed to minimize disturbance to bivalves’ behaviour, were made of two resin-coated electromagnets (56 mg each) glued on each valve and connected by 1.5 m flexible wires to a lab HFNI valvometer. An electromagnetic current between the electrodes was generated, allowing measurement of the amplitude of valve opening expressed from 0 to 100%. The laboratory protocol used here was designed to sample at 3.3 Hz from 16 animals in a sequential order. Every 300 ms, 3 packets of information were produced: distance between valves at the electrode level, sampling time and animal number. According to the present sampling rate, at the individual level, the system performed a measurement of the opening status every 4.8 sec. The basic principles are developed in Sow et al. [27], and examples of application are reviewed in Andrade et al. [28]. The data were analysed according to two protocols. They were transmitted via an acquisition card (NI-USB-6009, National Instrument, Austin, TX, USA) and recorded by a computer using in-house script in LabVIEW (National Instrument). They were also processed to a DELL workstation for analysis performed automatically using both a Bash script (Unix) and mathematical code written in R. The original records were published online on the professional pages of the MolluSCAN eye website (https://molluscan-eye.epoc.u-bordeaux.fr/index.php?rubrique=accueil&lang=en&site=EYRAC).

An individual response was characterized by a decrease of valve opening amplitude (VOA) as shown in Fig 1D. The amplitude of the response, expressed as a percentage, was calculated as

$$\Delta y=\frac{y_{1}-y_{2}}{y_{\text{max}}-y_{\text{min}}}\times 100$$ (1)

where y_1_ is the distance between the electrodes at the beginning of the change of VOA; y_min_, the daily minimal distance between electrodes (valve closed); y_2_, the minimal distance between the electrodes during the decrease of VOA; y_max_, the daily maximal distance between electrodes (valve fully open). The response, a decrease of VOA, was considered a response to sound if it appeared in the 70 sec fade-in stage of the sound exposure and if its amplitude was at least –10%. For each replicate, the number of responses was counted at each frequency and expressed as percentages. In addition, for every sound test, the sound pressure level and the corresponding shell acceleration at which the valve closure started were noted during post-analyses. Three different parameters were derived from this primary endpoint. They were the percentage of responding individuals, the amplitude of valve closure and the delay of the response.

### Statistics and estimation of sound perception sensitivity (threshold curves)

As stated above, oysters were exposed to different sound frequencies with t_0_ representing the beginning of the test. When a response occurred, the response delay, the associated pressure level and the shell acceleration were computed. To describe the effect that the explanatory variables (SPL and acceleration) has on the dependent variable (% of response in the group), linear regression is the most frequently used type of regression in predictive analysis. However, when the response takes one of only two possible values representing a presence or absence of reaction (0 or 1), the appropriate regression analysis is logistic regression. Logistic regression is used to explain the relationship between a dependent binary variable and one or more independent variables. The specific form of the model is as follows:

$$logit(p)=\text{log}\left(\frac{\pi }{1-\pi }\right)=\beta_{0}+\beta_{1}\text{log}(x)+\varepsilon$$ (2)

where *β*_0_ is the intercept parameter and *β*_1_ is the vector of the slope parameter. They are unknown regression parameters to be estimated. Maximum likelihood estimators (MLEs) were considered. The principle of MLE is to find estimators that maximize the likelihood function. The estimated parameters of the model were used to predict the percentage of response in the group relative to sound pressure level and/or shell acceleration. This allowed us to produce a predicted behavioural threshold curves that summarizes the sensitivity of oysters at different frequencies. To assess the goodness of fit of the model and how well the dependent variable is predicted based on the independent variables, the Hosmer-Lemeshow test [29] and likelihood ratio index [30] were used. Comparisons among frequency groups were performed by analysis of variance (ANOVA), after checking assumptions of independence, normality and homoscedasticity of the data. When the normality and the homoscedasticity were not met graphically and from ad-hoc tests, we used a Kruskall-Wallis test. If significant effects were detected, the multiple comparison tests between treatments was used to determine which groups are different with pairwise comparisons adjusted appropriately. For all statistical results, a probability of p < 0.05 was considered significant. All data are provided as supporting Informations (S1 Database)

## Results

### First delimitation of the sound perception range

As a preliminary experiment, oysters were exposed to a pair of wide-range acoustic sweep tones at maximum acoustic energy. A decrease of valve opening amplitude (VOA, Fig 2B) was observed in the oysters for the sweep composed of frequencies ranging from 10 to 600 Hz with an averaged pressure level of 146 dBrms re 1 μPa and a shell acceleration of 0.4 m∙s^-2^. No reaction was seen when oysters were exposed to the sweep of frequencies from 1 to 20 kHz with an averaged pressure level of 148 dBrms re 1 μPa and a shell acceleration of 0.1 m∙s^-2^. Based on this observation, our study focused on their sound perception responses to tones in the frequency range from 10 to 600 Hz.

### Typical responses to sound

Depending on the sound frequency, the number of responding oysters exhibiting a decrease of VOA ranged from 0 to 100%, and the valve closure amplitude and the response delay were variable. Fig 2B shows a typical set of VOA changes in 4 oysters responding to a 3 min tone at 100 Hz, 1.32 m∙s^-2^ at shell level and 158 dBrms re 1 μPa. All valve closures started less than 5 seconds after t_0_. The maximum VOA decrease was reached in approximately 30–40 sec. This was systematically followed by a slow valve reopening starting either during or after the 3 min sound test. Then, the valve opening amplitude remained steady, without any particular agitation, until the next sound test. Similar responses were never observed when the sound was turn-off. In a preliminary test series, it was found that the responses were independent of the order of sound frequency presentation, either decreasing or increasing (p < 0.05). We then turned to an analysis of the frequencies oyster can perceive at maximum acoustic energy before looking for their hearing thresholds.

### Hearing range

**Fig 3:**
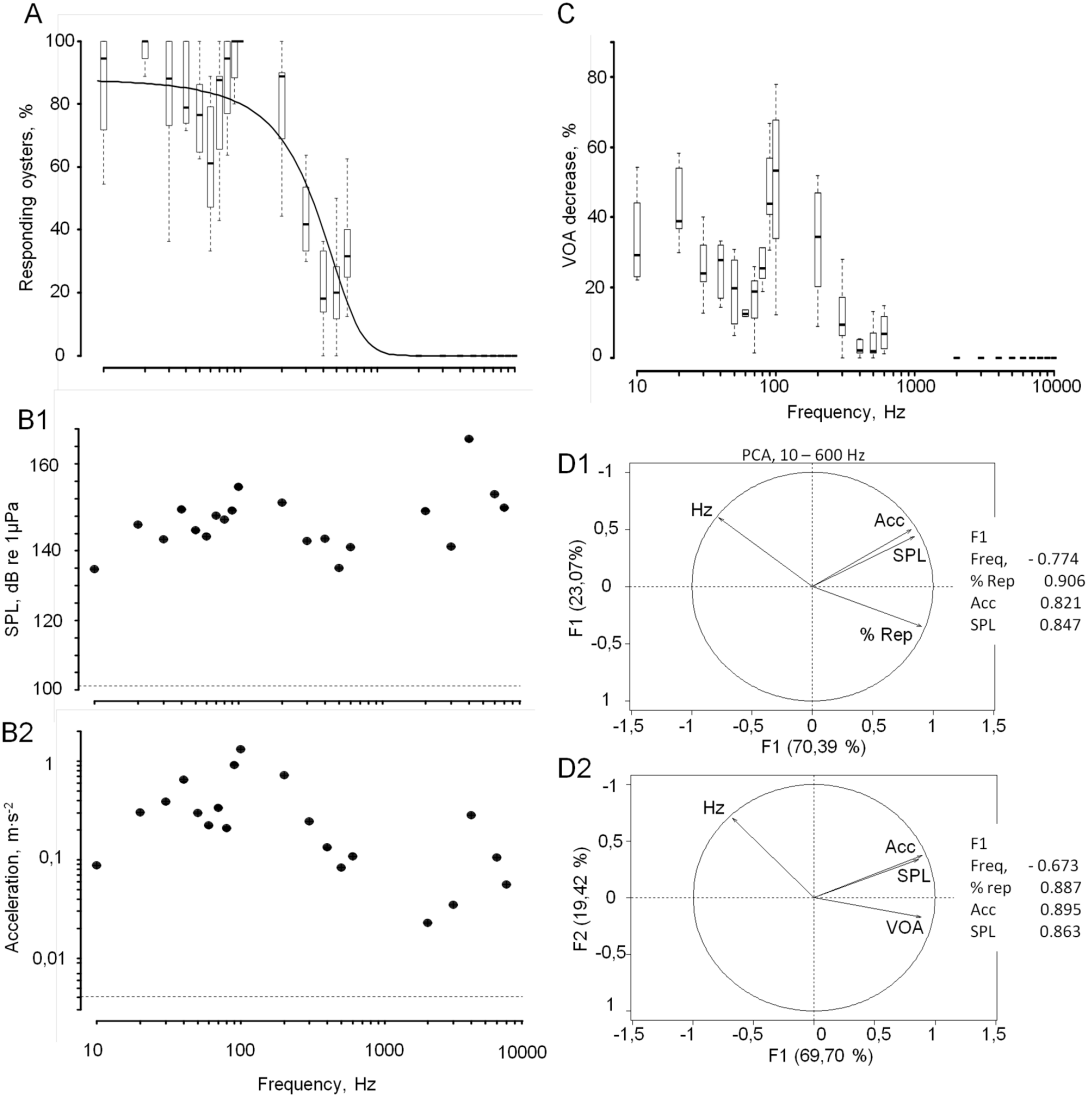
Oysters responded to sound frequencies and exhibit two peaks of maximum sensitivity at 20 and 90–100 Hz. A, a logistic regression described the relationship between the percentage of responding oysters in a group and sound frequency. For each frequency, the distribution is described by quartiles (bold line, median). B1, the measured sound pressure level, SPL, for the studied frequencies expressed in rms. B2, the measured shell accelerations at various frequencies expressed in rms. C, the relationship between the percentage of valve opening amplitude and sound frequency. At each frequency, the data distribution is described by quartiles. N = 16 oysters. D1 and D2, Principal Component Analysis describing the correlation between the percentage of responding oysters (% Rep, D1) and valve opening-amplitude decrease (VOA, D2) combined with frequency (Hz), shell acceleration (Acc) and sound pressure level (SPL).

The calculated response curve shown in Fig 3A shows that oysters were sensitive to frequencies ranging from 10 to <1000 Hz. The greatest response falls within the frequency range 10–200 Hz, with 60–95% of animals responding during each test. An analytical approach describing the response curve by logistic regression highlights the highly significant correlation between response and sound frequency (% of responding oysters = 2.021–0.006 frequency; p < 0.001). The percentage of responding oysters to frequencies from 10–40 and 90–100 Hz was significantly different to response from 300–600 Hz (p < 0.05). As loudspeakers do not produce a constant maximum acoustic energy at all frequencies, Fig 3B1 and 3B2 show respectively the simultaneously measured SPL in the water column (Fig 3B1) and the corresponding maximum shell acceleration (Fig 3B2) from 10–10 000 Hz. It shows that SPL was independent of frequency (r^2^ = 0.06, p = 0.13) while some lowest shell accelerations were measured at the highest frequencies (r^2^ = 0.12, p = 0.06).

We then switched to an analysis of the change of valve opening amplitude at these various sound frequencies to broaden our view of the oyster’s sensations. Specifically, we examined whether the change of VOA revealed a protective behaviour or a stress reaction that was related or proportional in some way to sound frequency. Fig 3C shows that two frequency ranges, 10–20 and 90–100 Hz, induced a maximum decrease of VOA of 43 ± 3 and 51 ± 6%, respectively. In contrast, the frequencies near 60 Hz (40–80 Hz), which were already associated with a significantly lower percentage of responses in the group (see Fig 3A), induced a weakest decrease of valve opening amplitude, with a mean value of only 12 ± 0.7%. This minimal decrease was not different from the closing amplitude observed at 300 and 600 Hz (12 ± 2% at 300 Hz, p = 0.868 and 9 ± 3% at 600 Hz, p = 0.062) but significantly different from the responses at 20 and 100 Hz (p < 0.01). To quantify the above observations, Principal Component Analysis (PCA) were performed on the percentage of responding oysters and valve opening-amplitude decrease (VOA) combined with frequencies ranging from 10–600 Hz (the hearing range), shell accelerations and sound pressure levels (Fig 3D1 and 3D2). Components 1 and 2 explained ≈ 90% of the total variance (Fig 3D1, 93.46%; Fig 3D2, 89.12%). Fig 3D1 and 3D2 show that the percentage of responding oysters and the valve opening amplitude were positively correlated to shell acceleration and sound pressure levels. On the contrary they were negatively related to frequency.

**Fig 4:**
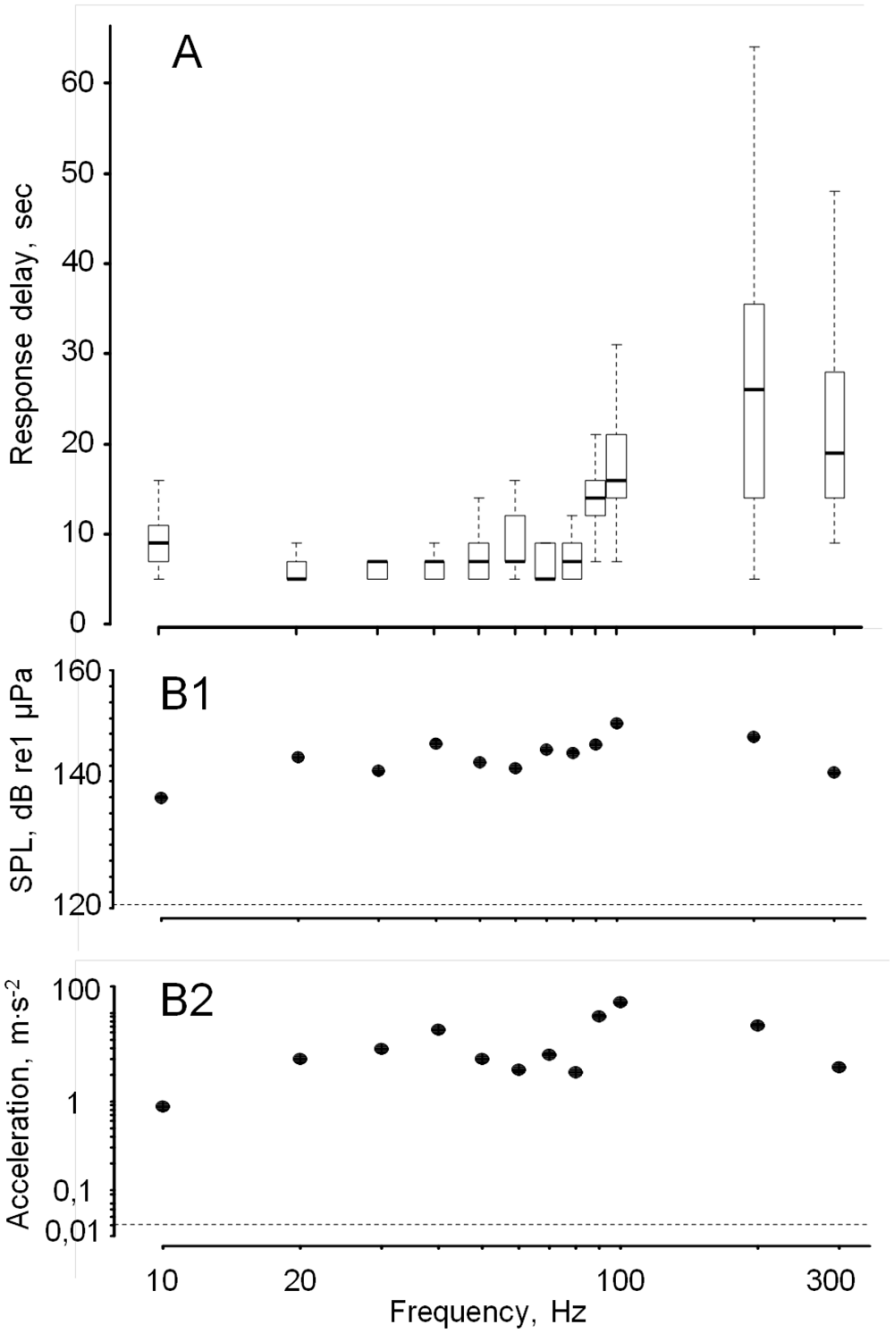
Response delay is function of sound frequency from 10–300 Hz. A, The response delay was systematically shorter from 10–80 Hz and the variability smaller from 10–80 Hz, with an exception at 60 Hz, illustrating a particular sensitivity to the lowest frequencies, N = 16 oysters. B1 and B2 represent the measured sound pressure levels, SPL, and shell accelerations for frequencies from 10–300 Hz.

To gain a final insight into the responses of oysters to various frequencies, we next examined the response delay of each oyster with respect to the stimulus onset (with t_0_ representing the time at which the fade-in period of the sound test started). The delay and the response delay homogeneity varied with frequency (Fig 4A). A multiple regression with “forward selection” showed that frequency explains 75% of the variability of response delay, while shell acceleration and pressure level respectively explain 10% and 1% of the variability. The delays were systematically shorter and more homogenous for frequencies ranging from 10 to 80 Hz. They were longer and more variable for frequencies from 100 to 300 Hz. The response delay at 90 Hz was longer than the delay at 10, 20, 30, 40, 50, 70, 80 Hz (p < 0.05), whereas it was not significantly different to 60 Hz. As the valve closure is a result of sound perception by the animals, it shows that low frequencies carried a different meaning than higher frequencies. To continue our examination of oyster sound perception, we then switched to an analysis focusing on their sensitivity to sound intensities.

### Sensitivity to sound intensity: Towards a graphic representation

**Fig 5:**
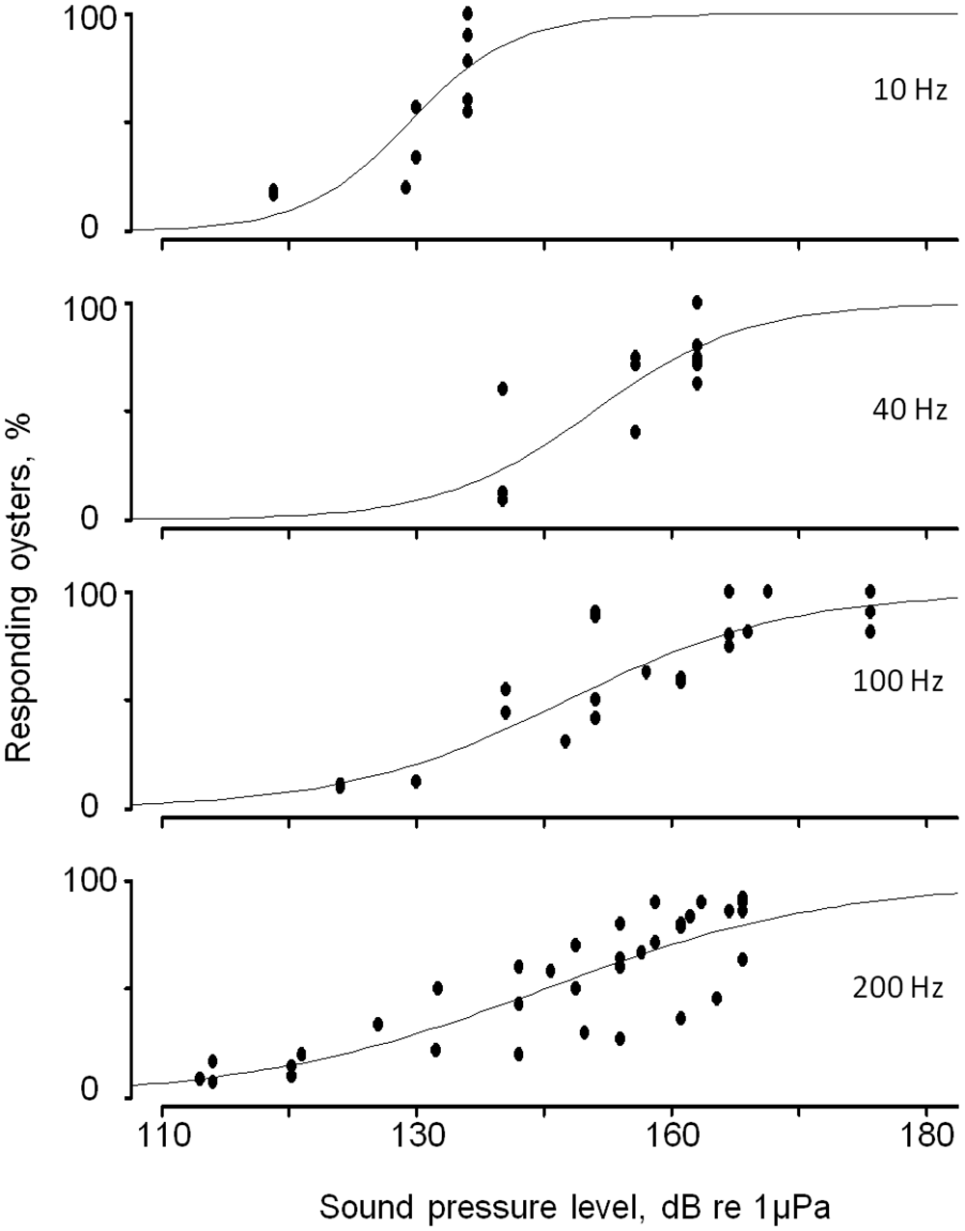
Identifying thresholds at various sound pressure levels for various frequencies. Four examples of logistic regression models describing the relationship between oyster group responses and sound pressure levels at 10, 40, 100 and 200 Hz. Sound pressure levels are expressed as dBrms.

The minimum sound intensity associated with a decrease of valve opening amplitude was studied at various frequencies (10, 30, 80, 100, 200, 300 and 400 Hz). The dependent variable was the percentage of oysters showing a decrease of VOA. A logistic regression was performed to estimate a model that adequately explained the relationship between valve response and acoustic energy. Fig 5 presents 4 examples of response curves for 10, 40, 100 and 200 Hz. The model was significant for frequencies ranging from 10 to 400 Hz but not significant for 500 or 600 Hz. Consequently, these frequencies were not included in the estimation.

**Fig 6:**
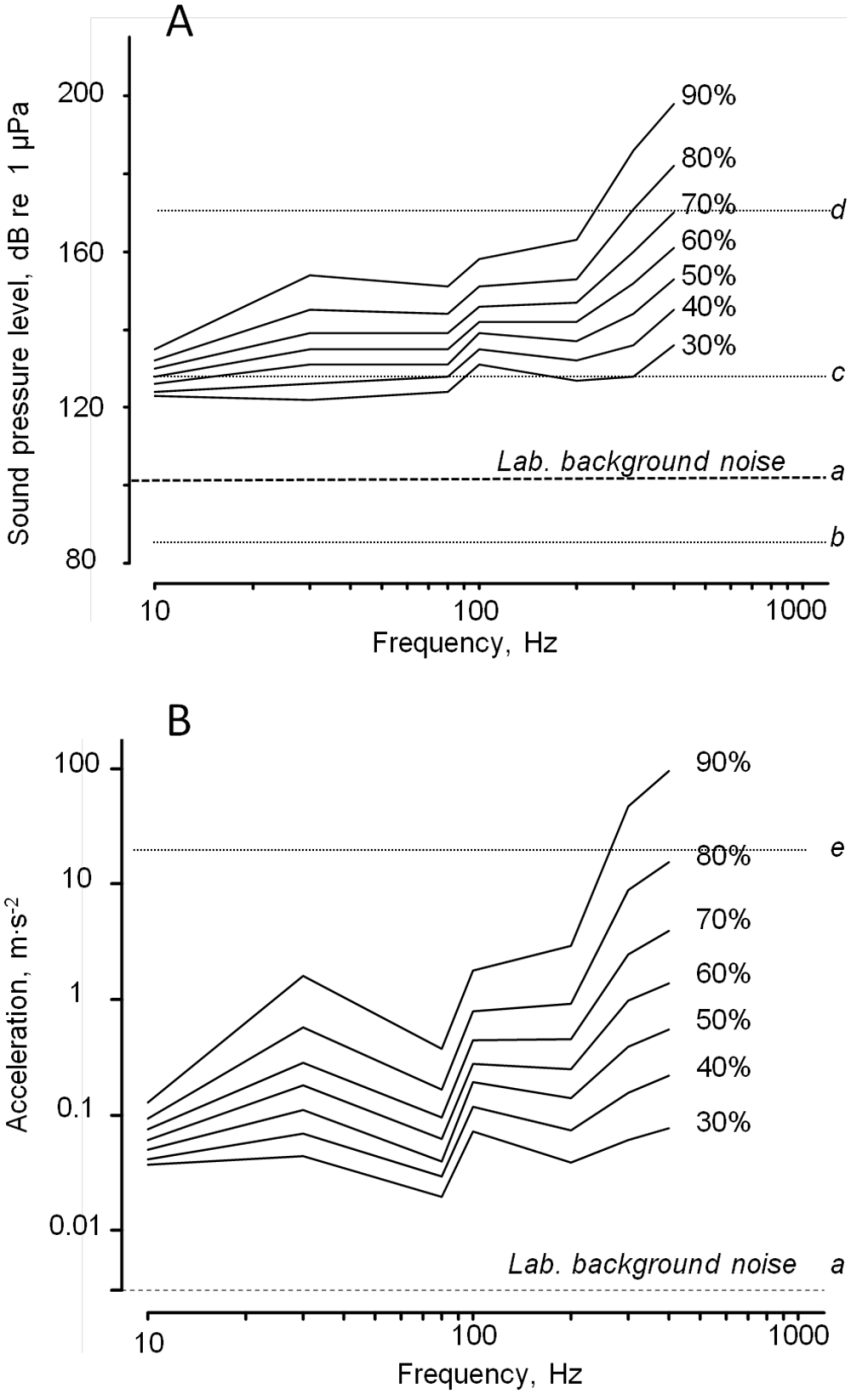
Behavioral thresholds (*M*. *gigas*) based on the percentage of responding oysters in a group. At each sound frequency, the percentage of responding oysters increased with sound pressure level, SPL, and shell acceleration allowing a family of curves to be drawn. A, Relationship among SPL, frequency and percentage of responding oysters. a, b, c and d, are examples of *in situ* noise recordings (rms; *a*, background noise under laboratory conditions; *b*, background noise at Eyrac pier, Bay of Arcachon, France; *c*, background noise in Poole Harbour, UK [31]; *d*, noise level produced by a cargo boat at 10 m away [32]. B, Relationship among shell acceleration, frequency and percentage of responding oysters. *a*, laboratory background noise; *e*, water motion of breaking waves on a rocky shore [33]. The minimum acoustic energy required to evoke a response increased with frequency.

Using the model, sensitivity thresholds to acoustic energy at various frequencies were obtained. The acoustic energy levels required to induce a valve reaction in 30 to 100% of individuals in the exposed oyster groups (effect acoustic energy, EAE, from 30–100%, EAE_30_-EAE_100_) were estimated. Fig 6 shows the corresponding series of curves including sound pressure levels (Fig 6A) and shell accelerations (Fig 6B). Clearly, the acoustic energy required to obtain a given percentage of response increases with frequency. Oysters are the most sensitive to sound at the lowest frequencies. To support the interpretation, we added informations to the graph about various marine noise backgrounds. On Fig 6A and 6B, the dashed line *a* shows the background noise in our laboratory conditions. On Fig 6A dashed line *b* is a noise recorded in the Bay of Arcachon in winter (Eyrac Pier, France) with no boating around, the dashed line *c* shows the background noise for Poole Harbour (UK, [31]) and the dashed line *d* gives the sound level (172 dB re 1 μPa) produced by a cargo vessel at 10 m (173 m length, 16 knots; [32]). On Fig 6B, the dashed line *e* shows an example of flow acceleration generated by breaking waves on rocky shores, 2 cm above the substratum, at Pacific Grove, California (within beds of the mussel *M*. *californianus*; wave height, 0–0.5 m; [33]).

## Discussion

The present work provides a study of sound perception ability in oysters *M*. *gigas* using a behavioural approach based on transient valve closure reactions. We have shown that *M*. *gigas* is sensitive to sound in the range of 10 to < 1000 Hz—independent or nearly independent of sound pressure level and shell acceleration under our experimental conditions—with two maximum valve closure reactions at 10–20 Hz and 90–100 Hz. T**h**e response delay was systematically faster in the lowest frequency range, 10–80 Hz. The minimum acoustic energy required from 10–80 Hz to evoke a response in 30% of a group was estimated by modelling to ≈ 122 dBrms re 1 μPa and 0.02 m∙s^-2^. It increased with frequency. Importantly, as a partial valve closure cannot be differentiated from a response to a nociceptive stimulus, the data show that *M*. *gigas* should detect lower acoustic energy.

### Comparison with published data in terms of bandwidth and acoustic energy

This is the first report quantifying the sound perception ability in oysters. While a large amount of work has been devoted to fish [20], very few works have focused on the question of sound perception in bivalve molluscs. Most likely, the most advanced one is the work by Roberts et al. [16] on blue mussels, *M*. *edulis*. As we did in our study, they used valve reaction as an endpoint of sound perception. They studied the impact of substrate-borne vibrations—mimicking anthropogenic operations such as pile driving and blasting—while we studied the perception of sound generated in the water column. Using a shaker system, Roberts et al. [16] produced pure tones in the range of 5–410 Hz, and they established the threshold sensitivity of mussels lying on a sand and gravel bottom. They concluded that *M*. *edulis* responses were relatively constant from 5–90 Hz with a sharp decrease in sensitivity at 210 Hz. Under their experimental procedure, the minimum sound intensity required to initiate a response ranged from 0.06 to 0.55 m∙s^-2^ (rms, 5–410 Hz). Although there are differences of experimental design and possibly species-specific differences, these findings are consistent with present results. On another side, unlike what they observed, oysters in the present work exhibited a gradual and continuous reduction in sensitivity with increasing frequency above 200 Hz while for mussel thresholds were relatively constant across frequency.

Mosher [13] was probably the first to demonstrate that a bivalve mollusc can detect low frequency vibrations. He elicited burrowing behaviours in *Macoma balthica* exposed to vibrations produced by a small solenoid unit working at 2–50 Hz (unknown SPL) and applied on the aquarium sides. The activity was recorded by a kymograph, and the response was obtained after a minimum of 5 sec exposure, which is comparable to the minimum response delay reported in the present work. Ellers [14] studied the swatch–riding clam *Donax variabilis*. *D*. *variabilis* perceive the vibrations created by incoming waves at frequencies ranging from 1 to 1000 Hz. They were more responsive to frequencies from 20–140 Hz (at 146 dBrms re 1 μPa) and pure tones at 72 Hz (150 dBrms re 1 μPa). They were less responsive to 832 Hz despite a relatively higher pressure level (166 dBrms re 1 μPa). Using a different approach, Zhadan [15] studied the role of the abdominal sense organ (ASO) in two pecten species, *Mizuopecten yessoensis* and *Chlamys swifti*. He reported that contractions of the mantle velum edge in both species are sensitive to modulated ultrasonic vibrations in the range 30–1000 Hz without any information on applied sound pressure level. Finally, Vazzana et al. [17] studied behaviour and changes of haemolymphatic parameters in *M*. *galloprovincialis* following 30 min exposures to various sweep tones ranging from 100 Hz to 60 kHz (maximum SPL of 150 dBrms re 1 μPa). They observed no change of behaviour whatever the applied sound frequency. At 100–5000 Hz, which includes frequencies to which invertebrates are responsive, they found significantly higher values of the following biochemical stress parameters: glucose, total protein, total haemocyte number, HSP 70, and AChE activity. These results show that the internal physiology of *M*. *galloprovincialis* can be significantly modified by frequencies similar to what was reported for *M*. *edulis* [16] and what we report here for *M*. *gigas*.

In Mollusca, a significant amount of work has been devoted to cephalopods. *Octopus ocellatus* [10] is remarkably sensitive to frequencies ranging from 50 to 280 Hz with a minimal threshold at 0.0005 m∙s^-2^ (rms). As in oysters it exhibits a decrease of sensitivity above 200 Hz. The cuttlefish *Sepia officinalis* have been reported to react to frequencies from 1–100 Hz with a threshold ranging from 0.008 to 2 m∙s^-2^ (rms; [12]). In the same species, Samson et al. [34] studied the relationship between sound pressure levels, frequency and different types of behavioural responses. They exposed them to pure tones ranging from 80 to 1000 Hz with SPL of 85–188 dBrms re 1 μPa and particle acceleration from 0–17.1 m∙s^-2^. It is worth noticing that the first reported behavioural changes (small body pattern change and fin movements) occurred at 150 Hz and 0.02 m∙s^-2^ (125 dBrms re 1 μPa), which is comparable to the sensitivity reported at 200 Hz for *M*. *gigas* (0.04 m∙s^-2^ and 128 dBrms re 1 μPa; Fig 6).

In crustaceans, Roberts et al. [7] reported sensitivity ranging from 0.02–0.44 m∙s^-2^ (rms, 5–410 Hz) for *Pagurus bernhardus* exposed to substrate-borne vibrations. Salmon [8] showed that *Uca pugilator* is more sensitive to frequencies ranging from 30 to 60 Hz with a threshold of 0.04–0.05 m∙s^-2^ (rms). It is less sensitive to frequencies between 240 and 1000 Hz (2 m∙s^-2^, rms). The frequency range and highest sensitivity reported in crustaceans are then close to values found in oysters (current work) and mussels [16].

On the contrary fish without swim bladder appears to be more sensitive to sound than oysters. Karlsen [35] examined the sensitivity of the plaice *Pleuronectes platessa*. He measured thresholds of approximatively 0.00005 m∙s^-2^ (rms) at 10 and 30 Hz. The cod *Gadus morhua* is even more sensitive at 60 Hz with a threshold reaching 0.00001 m∙s^-2^ (rms; from [36]).

### How do oysters perceive sound vibrations?

In water-breathers, sound reaches sense organ directly since their body has more or less the same density as the water. Their sense organ, along with the rest of the body, moves with the sound. Different structures allow such a perception of sound pressure and/or of particle motion [6, 37]. In fish, the swim bladder or any other gas-filled structure gives sound pressure sensitivity. In the absence of a gas-filled structure, as, for example, in elasmobranch, the otolith organs allow sound detection through direct response to acoustic particle motion [38]. In crustaceans, different sensory systems are present including setae cells, chordotonal organs and statocyst receptors [6, 39]. In molluscs, including bivalve molluscs, statocysts also exist, and since they are mass loaded by statolith(s) they are evidently good candidates to detect particle motion in a similar manner to the otolith-loaded hair cells in the vertebrate ear. In bivalve molluscs, statocysts and hair cells have been described in adults and larvae of scallops (*Pecten maximus*), blue mussel (*M*. *edulis*), freshwater mussels (*Anodonta cygnea*) [39, 40, 41], Anomalodesmata (which include the razor shells [42]) and oysters (*Ostrea edulis* [43]; Tsirulis [44] cited in Budelmann [45]).

In cephalopods, the role of statocysts has also been well studied. As reviewed by Budelmann [45], cephalopods have been shown to detect water-borne sounds using their statocysts. Williamson [46] showed that isolated statocysts of octopus showed peak sensitivity at 70–100 Hz, and Kaifu et al. [10] reported that an absence of statocyst abolishes the response following behavioural tests. Cephalopods have also superficial receptor systems that sense local water pressure movements. They are analogous to the amphibian and fish lateral line and include the presence of ciliated sensory cells. They are highly sensitive to local water oscillation in the range of 0.5–400 Hz, with a threshold at 100 Hz as low as 0.06 μm peak-to-peak water displacement at the receptor cell [45].

### What do oysters perceive in the field?

To review the various vibrations present around oyster *M*. *gigas* fields, one can differentiate between biotic and abiotic vibrations of natural and anthropogenic origins. In shallow waters, the main abiotic source is certainly the breaking surf, emitting at frequencies from 10–800 Hz and at sound pressure levels up to 120 dBrms re 1 μPa between 10 and 20 Hz [47]. Records made from the pier at Scripps Institution of Oceanography (California, US) report breaking waves generating sound pressure levels up to 110–118 dB re 1 μPa between 100 and 200 Hz [48]. Gaylord [33] studied the relationship between wave height, intertidal water velocities and accelerations on rocky shores. He reported that fully breaking waves 0–0.5 m high produced 1.5 m∙s^-1^ velocities and 25 m∙s^-2^ accelerations while 1.0–1.5 m high waves generated 3.5 m∙s^-1^ and 70 m∙s^-2^ velocities and accelerations. Water flow and currents also emit sounds that are interesting for the present purpose. Tonolla et al. [49] reported that a current of 0.4 m s^-1^ can generate noise at frequencies from 30 to 500 Hz and with a sound pressure level up to 120 dB, and they suggested that it could affect fish behaviour, triggering displacement to different habitats. Mat et al. [50] reported that the tidal rhythmicity of oyster valve closing/opening, described by Tran et al. [21] in the coastal zones, follows either an endogenous or an exogenous pathway, by means of a circadian clock or not. We show that sounds and vibrations produced by breaking waves and currents are in the sensitivity range of mussels and oysters. We propose that, at rising tide it could be a tidal cue triggering oyster circatidal activity and increasing their fitness to their particular habitat. Ubertini et al. [51] reported that thunderstorms could be potential spawning triggers in the oyster *M*. *gigas* and that lightning impacts are associated with the largest larvae cohorts in the Thau lagoon (France). Thunderstorms include claps of thunder and produce infrasound bursts that are 3 sec in duration with peaks at 10–30 Hz and 100–300 Hz [52] which are audible to oysters. Consequently, we also propose that the hearing ability in oysters could (i), play a role in synchronizing spawning events, (ii) influence spawning efficiency and then (iii) participate indirectly in the control of oyster population dynamics.

Numerous animals produce sounds at frequencies noticeable by oysters or other bivalves that have the same capacity. The limiting factors will be the acoustic energy and the distance from the source. Interestingly, numerous fishes produce sounds below 1 kHz. The oyster toadfish, a well-known predator of young oysters, produces an agonistic sound with a fundamental frequency between 90 and 100 Hz [53]. Swimming fishes produce sounds mainly composed of frequencies less than 100 Hz [54], which are, again, in the oyster hearing range. Lobsters, *Homarus americanus*, which can feed on young oysters produce carapace vibrations from 90–260 Hz, which generate water-borne acoustic signals [55]. In the tropics, sounds produced by crustaceans have been recorded. Patek and Cadwell [56] reported that the mantis shrimp, *Hemisquilla californiensis*, known to kill prey such as sea snails by spearing and stunning them, produces sounds at a fundamental frequency ranging from 20 to 60 Hz. Fiddler crabs, which can also feed on young oysters, found among mangroves and along sea beaches, lagoons and swamps, produce sounds correlated with the vibratory movements of their ambulatories. Part of this sound profile is at frequencies between 150 and 250 Hz. These sounds are directly transmitted in the substrate, and the louder sounds can propagate at distances of 10 m [57]. On a theoretical basis, all of the above sounds and vibrations could be perceived by oysters. In contrast, they should not hear snapping shrimps, the major source of biological noise in coastal tropical waters (peak-to-peak source levels varying from 183–189 dB re 1 μPa [58]), because they produce frequencies in the range 2–200 kHz. This of course is speculation, but it does raise the prospect of exciting research directions for the future.

For the last few decades, noise pollution has been a major problem in the marine environment, evidently including the coastal zone inhabited by oysters and various bivalve molluscs. Most of this pollution is at low frequencies (below 1 kHz) and is due to distant and nearby cargo boats scattered in the oceans (for recent reviews, see [32, 59]). Shipping generates sounds up to 180–190 dB re 1 μPa @ 1 m, with much of the power concentrated below 200 Hz. However, other sources are also of major importance. The most powerful are explosions (up to 300 dB re 1 μPa @ 1 m for 10–200 Hz) and seismic research (220–230 dB_peak_ re 1 μPa for 5–300 Hz; and vibrations level up to 0.001 m∙s^-1^ peak at 296 m from the source). Pile driving can generate up to 237 dB re 1 μPa @ 1 m for 20–1000 Hz, drilling from a fixed platform produced noise up to 145–190 dB re 1 μPa @ 1 m at frequencies between 10 to 100 Hz, and wind turbines generate sound up to 140–150 dB re 1 μPa @ 1 m in the bandwidth 16 Hz to 20 kHz with a major amplitude at 30–200 Hz [32]. Importantly, note that shipping and wind turbines are considered to produce sound continuously. All of these sounds are within the hearing ability of oysters especially if we consider that substrate vibration is also encountered as a result of sound in the water. In contrast, small recreational boats, jet skis and water bikes, which produce sounds at 1–5 kHz (150–180 dB re 1 μPa @ 1 m), should not be heard by oysters.

Invertebrates are a major component of biodiversity. Understanding their relationship with the world of sound, especially how they interact with it, is clearly under evaluated but is a fascinating challenge.

## Supporting information

S1 Database All data base. (XLSX)
